# Spontaneous Emergence, Imitation and Spread of Alternative Foraging Techniques among Groups of Vervet Monkeys

**DOI:** 10.1371/journal.pone.0047008

**Published:** 2012-10-10

**Authors:** Erica van de Waal, Andrew Whiten

**Affiliations:** 1 Centre for Social Learning and Cognitive Evolution, and Scottish Primate Research Group, University of St Andrews, School of Psychology, St Andrews, United Kingdom; 2 Inkawu Vervet Project, Mawana Game Reserve, Swart Mfolozi, KwaZulu Natal, South Africa; Université Paris 13, France

## Abstract

Animal social learning has become a subject of broad interest, but demonstrations of bodily imitation in animals remain rare. Based on Voelkl and Huber's study of imitation by marmosets, we tested four groups of semi-captive vervet monkeys presented with food in modified film canisters (“aethipops’). One individual was trained to take the tops off canisters in each group and demonstrated five openings to them. In three groups these models used their mouth to remove the lid, but in one of the groups the model also spontaneously pulled ropes on a canister to open it. In the last group the model preferred to remove the lid with her hands. Following these spontaneous differentiations of foraging techniques in the models, we observed the techniques used by the other group members to open the canisters. We found that mouth opening was the most common technique overall, but the rope and hands methods were used significantly more in groups they were demonstrated in than in groups where they were not. Our results show bodily matching that is conventionally described as imitation. We discuss the relevance of these findings to discoveries about mirror neurons, and implications of the identity of the model for social transmission.

## Introduction

Social learning has been defined as “learning that is influenced by observation of, or interaction with, another animal (typically a conspecific) or its products” [2]. Such learning from others has increasingly been recognized to be a widespread phenomenon in the animal kingdom, often providing animals with an efficient source of information that can shape adaptive responses in such crucial domains as foraging, mate choice and predator avoidance [3]–[6]. Such processes have by now become extensively researched in primates [7], other mammals [8], birds [9], fish [10] and invertebrates [11]. Particular interest has focused on imitation – “learning an act from seeing it done” [12] – because of both the presumed cognitive specialization needed to translate perception of novel actions done by others into the performance of matching actions by oneself [13] and the hypothesis that the fidelity of copying offered by imitation is necessary for a species to exhibit substantial cultural transmission and in particular, cumulative cultural evolution [14].

Identification of imitation in non-human species has proved methodologically challenging. A common problem is that social learning is often studied in relation to actions that manipulate objects in functional ways, such as in processing foods or using tools. In such contexts it is inherently difficult to distinguish imitation, the learning of actions (as defined by Thorndike) from learning about their environmental effects or the affordances of the objects manipulated through these actions. One solution to this dilemma has been to arrange ‘two-action’ experiments in which the same outcome is achieved by each of two models who use different actions to do this, particularly where this involves different body parts [15]. Imitation is then apparent when observers differentially match their own later actions to the action and body part variant they witnessed.

Such bodily imitation was demonstrated by Zentall et al. [15] and Akins and Zentall [16], who showed that pigeons and quail respectively would tend to match conspecific models' use of either pecking or stepping responses to operate a manipulandum to obtain food. Similarly, Voelkl and Huber [1] showed that common marmosets would match manual versus oral techniques that they had observed models to use in removing film canister lids to gain the food inside. Most recently, Buttelmann et al. [17] showed that chimpanzees would imitate human models using either their head, foot or bottom to operate a device, in the latter case by sitting on it. This corpus of two-action (and three-action) bodily imitation studies has evidently remained quite small, despite its distinctive power to demonstrate imitation. The studies have also remained restricted to the dyadic configuration in which a single observer watches a single model, so the relevance of the results for the wider phenomenon of cultural diffusion of an innovation across a group of animals has yet to be examined.

Here we extend this approach to the group level, examining the copying of different bodily techniques to open an experimental ‘artificial fruit’, and the potential spread of such techniques to create different incipient traditions in different groups. In this way, we have married the use of the two-action approach for testing bodily imitation as a mechanism, to its more recent use for tracking the spread of differential behavioural traditions [18], [19]. Like Voelkl and Huber [1] we relied on natural variation amongst the techniques applied by the first individuals to solve the tasks presented, in different groups.

## Materials and Methods

### (a) Ethical Statement

Our experiments were approved by the relevant local authority, Ezemvelo KZN Wildlife, South Africa; by the funder, Swiss National Science Foundation as well as the Ethics Committee of the School of Psychology, University of St-Andrews, UK. Our set-up involved some feeding competition. However, as we were mainly interested in individuals' first manipulation we offered multiple test items to minimise conflict. We also kept the amount of food relatively small (5 raisins, or 5 peanuts, or 1 fruit jelly depending on the group) both in the demonstration and experimental phases.

### (b) Study animals

Experiments were conducted by EW with the assistance of staff from the Inkawu Vervet Project (see acknowledgements) between December 2010 and August 2011. Four groups of captive vervet monkeys (*Chlorocebus aethiops*) were studied. Three groups (‘Debbie’, ‘Hammer’ and ‘Sturrell’) were housed at the Wild Animal Trauma Centre and Haven (WATCH) in Vryheid, KwaZulu-Natal, South Africa and one group (Lisa) was at Bambelela Wildlife Care, Limpopo, South Africa. Both centres play a key role in the rehabilitation and release of vervet monkeys (henceforth ‘vervets’) in South Africa. They are home to numerous groups of vervets at various stages of rehabilitation, and they have already released groups to the wild.

All participant monkeys lived in stable groups of 22 to 37 individuals, typically composed of one adult male with many adult females and juvenile and are summarized in Table 1. All groups were kept in conditions to prepare them to be released later. Individuals were recognizable from their faces and other features such as scars, fur colour and tail shape already documented by sanctuary staff. The hierarchy within each group was documented by sanctuary staff on the basis of the outcomes of conflicts between pairs of individuals and priority of access to food sources. Rank is typically stable between adult female vervets and given only one male per group, there were no changes in the hierarchies during the study. The enclosures at WATCH were enriched with grass, trees, and climbing structures, with a ground area of 80 m^2^ (Hammer), 130 m^2^ (Sturrell) and 420 m^2^ (Debbie) and a height of 3.2 m in all three enclosures. The enclosure of Lisa group at Bambelela consisted of a concrete floor and climbing structures, with a ground area of about 50 m^2^ and a height of 3 m.

**Table 1:** The composition of the study groups.

Group	AM	AF	J	Infant	Total	Model + technique
Hammer	0	5	10	7	22	Dom AF = 5 hands
Sturrell	1	5	20	11	37	Sub JM = 4 mouth + 1 rope
Debbie	1	1	20	3	25	Dom JF = 5 mouth
Lisa	1	2	16	8	27	Dom AF = 5 mouth

Males are scored as adults through size and testis bright colours, while females are scored as adults once they have given birth. Group members that did not fulfil these criteria were scored as juveniles if they were over one year old. The individuals under one year old were categorized as infants and were not included in our analyses. Identity of the model is showed in last column: hierarchical rank (Dom = dominant, Sub = subordinate), age (A = adult, J = juvenile) and sex (F = female, M = male).

### (c) Experimental procedures

**Figure 1:**
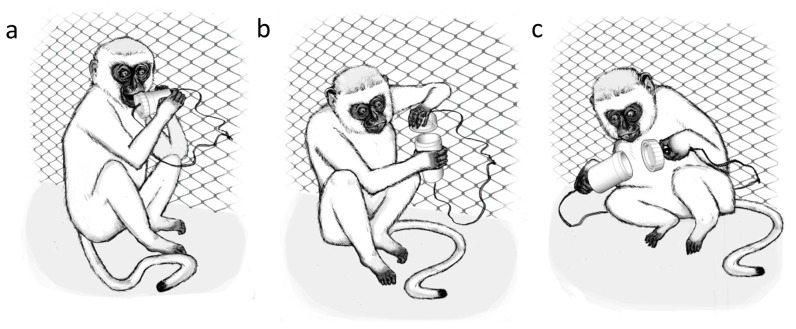
Alternative opening techniques. a) removing the lid with the mouth; b) removing the lid with the hand; c) pulling the ropes to open the aethipop.

The experimental apparatus consisted of a white, lidded cylinder 5.3 cm long and 3 cm in diameter, similar to a film canister (Fig. 1), with a food reward inside it (grapes, raisins, peanuts or fruit candies depending on the group), acting as an ‘artificial fruit’ [20]. The lid could be ‘popped’ off to gain the food inside. Noting the species' latin name we thus called this device an ‘aethipop’. Short lengths of rope were threaded through both the top and bottom of the tube to attach it to the monkey enclosures.

At WATCH, experiments took place in the entrances of the enclosures, which could be isolated from the main enclosures so the aethipops could be refilled with no monkeys present. These entrances were shaded by cloths to prevent monkeys from other groups observing the experiment.

The experimental procedure began with a step-wise training phase in which the individual most focused on the task would likely find an opening solution by trial-and-error. As each of the following four steps was completed by this monkey, the next was instituted until we had one proficient ‘model’ who opened a fully closed aethipop: (1) tube open; (2) lid just half on; (3) lid on but not fully closed; (4) lid closed. No particular technique was selectively encouraged. Once this individual discovered an opening technique, it was allowed to perform five openings, (‘demonstrations’) each time being provided with a single aethipop to ensure its exclusive access, with the remainder of the group being able to watch. Dominant females were preferred models, as van de Waal et al. [21] had found that in the wild, vervet females are watched and more likely to be learned from than males. In one group (Hammer) the dominant female could be comfortably separated in the entranceway such that others observed her five initial demonstrations through the mesh. In the other groups such separation was not possible, but fortunately dominant females self-selected to perform the five initial demonstrations in two other groups (Lisa and Debbie), while in the fourth group (Sturrell) this role was taken by a juvenile male. In all these cases, other monkeys were next to the model as they performed the initial five demonstrations. All models monopolised the apparatus and thus were the only group members manipulating the apparatus during demonstrations, prior to the experimental phase.

Fortunately, differentiation in the techniques used by the models emerged. The Hammer female consistently used her hands to open the aethipop, whereas the other two females exclusively used their mouths (Fig. 1a, Fig. 1b). The juvenile used his mouth also, but in his final demonstration he pulled the aethipop apart by grasping the ropes attached to lid and base and pulling in opposite directions (Fig. 1c). Thus, fortuitously, three techniques – ‘mouth’, ‘hand’ and ‘rope’ – were modelled in different groups.

After the demonstration phase, an experimental phase consisted of multiple trials in each of which many aethipops were offered, all tied to the mesh at about adult vervet eye level, but laid on the ground and at a distance of about 30 cm one from another. The three WATCH groups had five trials with 10 aethipops per trial. With the Bambelela group we had to conduct 10 trials with five aethipops per trial, to allow a staff member to refill all aethipops while being in the enclosure. This experimental setup provided a total of 50 openings per group, with all monkeys free to interact with the aethipops within the constraints of the social group dynamics, such as relative rank. All interactions with the aethipops were recorded using two video cameras.

### (d) Data collection, analyses and statistics

For each manipulation of an aethipop we coded from the video records which monkey performed, which technique it used (attempting to remove the lid with mouth, hand or by pulling the rope) and whether it managed successfully to remove the lid and gain the reward or not. Manipulations were called ‘attempts’ if they were not successful, and called ‘openings’ if they were successful and accessed the reward. Coding categories were first discussed between two coders, and then checked against video recordings. All codings were found to be unambiguous. Monkeys typically held the tube part of the aethipop in their hands, but opening involved either different body parts (mouth/hand) or different parts of the apparatus (lid/rope).

Individuals younger than one year never participated in the experiments. We investigated whether the three observed techniques (mouth, hand, rope) were used at similar frequencies by individuals of different groups, which technique group members used at first trial and the preferred technique used by each monkey during all their attempts and successful openings. The models in each group were of course excluded from these analyses. As opening using the mouth was the most common technique across all groups, we used it as the baseline measure against which to compare the occurrence of the rarer techniques (hands, ropes). All statistical analyses employed nonparametric tests using SPSS 17.0 (SPSS Inc., Chicago, IL, U.S.A.)

## Results

Over all 50 openings per group, a total of 51 monkeys made attempts (numbers in specific groups: D = 12, H = 12, L = 8, S = 19 monkeys) and 41 monkeys made more than one attempt. In total, 382 opening attempts were made (attempts in specific groups: D =  151, H = 95, L = 61, S = 75).

‘Mouth’ was observed to be the ‘default’ preferred approach insofar as all participating individuals attempted this technique at some time and all successful individuals used this technique at least once. Of interest was thus whether the rarer ‘hands’ and ‘rope’ techniques occurred preferentially in the groups in which models had begun using them.

### (a) Hand opening technique

**Figure 2:**
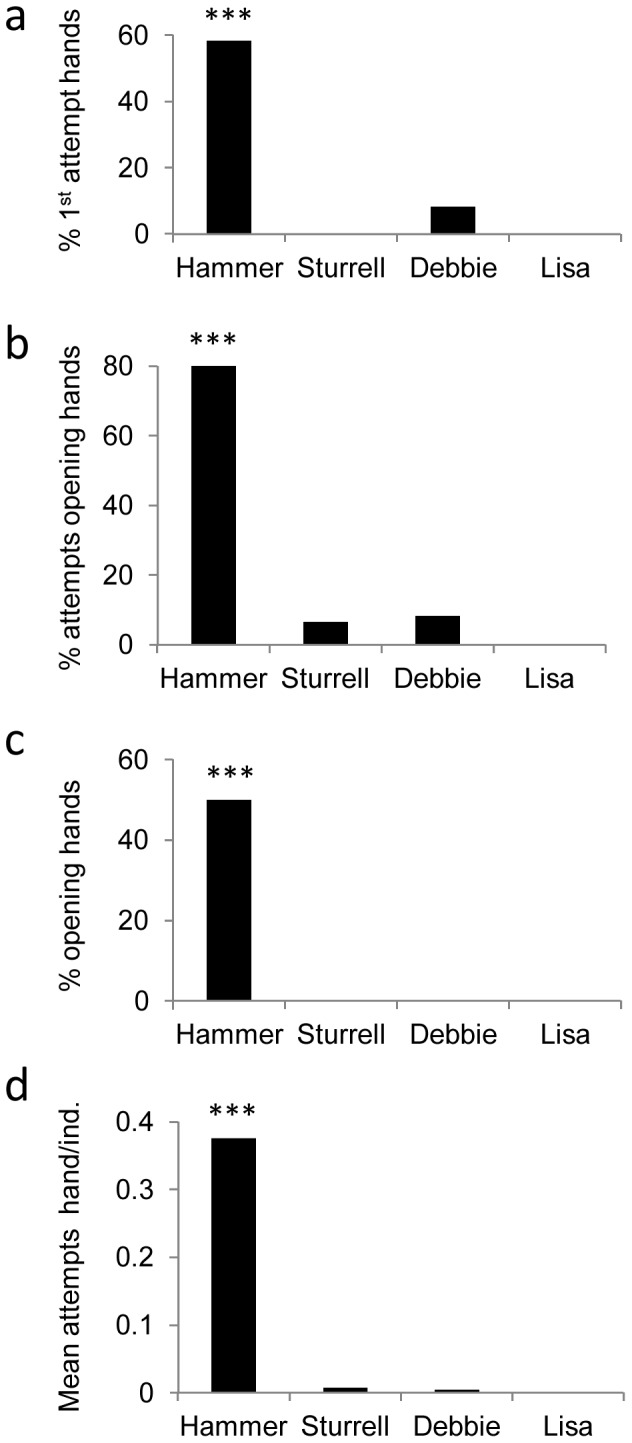
The use of the hand technique in each group. a) percentage of participating monkeys attempting to open with hand at first trial; b) percentage of participating monkeys attempting to open with hand across all trials; c) percentage of successful monkeys opening with hand; d) mean proportion of attempts with hand (hand/hand+mouth) per participating individual. *** indicates Hammer scores significantly higher than all other groups, for each measure (see text for details).

In the Hammer group which had the hand opening model, this technique was used by a significantly greater proportion of monkeys at their first attempt (7/12) than in the 3 other groups (1/39): Fisher exact test, p<0.0001, Fig. 2a), and this use of the hand was also true across all attempts (10/12 monkeys versus 2/39: Fisher exact test, p<0.0001, Fig. 2b). Focussing on successes, a significantly greater number of successful individuals opened with their hands in this group (4/7) compared to the other groups, neither of which displayed any successful hand opening (0/21: Fisher exact test, p =  0.0002, Fig. 2c). We also compared the proportion of hand versus other techniques attempted and found that Hammer monkeys used a much higher proportion of hand techniques in their attempts (mean = 0.38) than those in the other 3 groups (mean<0.01), Kruskal-Wallis test: n = 51, p<0.001, Fig. 2d). Hammer monkeys displayed a higher proportion of hand techniques in their successful openings (0.23) than in the other 3 groups, who showed none (n = 28, p = 0.001).

### (b) Opening by rope-pulling

**Figure 3:**
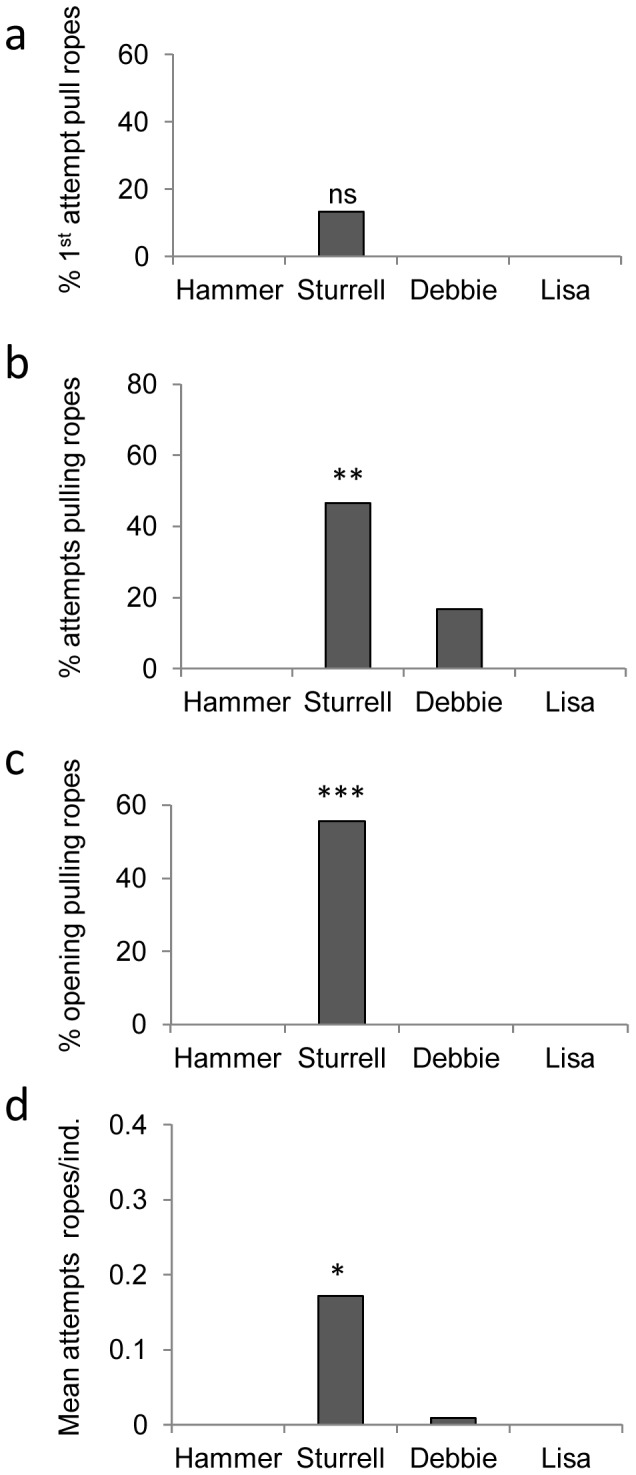
The use of the ropes technique in each group. a) percentage of participating monkeys attempting to open by pulling ropes at first trial; b) percentage of participating monkeys attempting to open by pulling ropes across all trials; c) percentage of successful monkeys opening by pulling ropes; d) mean proportion of attempts by pulling ropes (ropes/rope+mouth) per individual. *or ** or *** indicates Sturrell scores significantly higher than all other groups, for each measure (see text for details).

In the Sturrell group that witnessed the rope-pulling technique, we found that this technique was not used significantly more in first attempts (2/19) than in the 3 other groups (0/32) (Fisher exact test, p =  0.134, Fig. 3a). However significantly more monkeys attempted it in the Sturrell group (7/19) than the others (2/32) when all attempts were considered (Fisher exact test, p =  0.0099, Fig. 3b). Turning to successful openings, there was also a significantly greater number of successful individuals using the rope-pulling technique in this group (5/9) compared to the other groups, where none displayed it (0/19) (Fisher exact test, p =  0.0013, Fig. 3c). We also compared the proportion of the rope pulling versus other techniques and found that Sturrell monkeys used a much higher proportion of the rope pulling technique in attempts, successful or not (mean = 0.17) than in the other groups (mean<0.01) (Kruskal-Wallis test: attempts n = 51, p = 0.005, Fig. 3d). Sturrell monkeys also used a greater proportion of the rope-pulling technique in successful openings (0.25) than in the other groups, who did none (n = 28, p = 0.007).

### (c) Spread of successful openings

**Figure 4:**
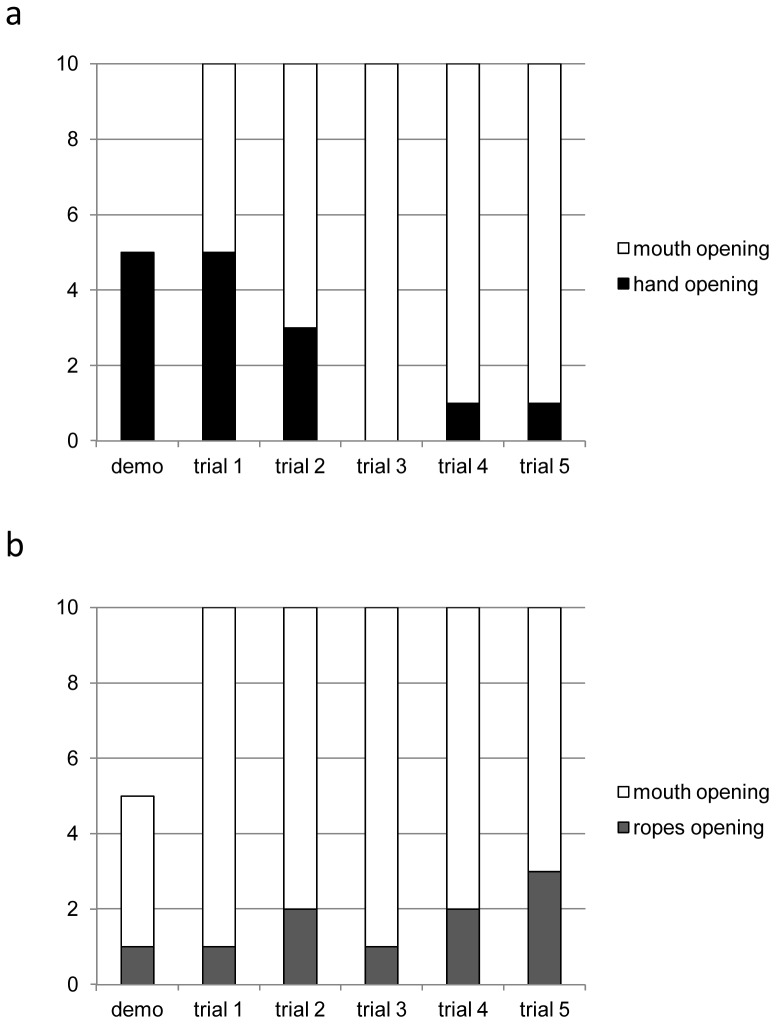
The spread across all trials of the rare opening techniques. a) successful hand opening in the Hammer group, b) successful opening by pulling ropes in Sturrell group.

The occurrence of successful hand openings across trials in the Hammer group is shown in Figure 4a. The spread of the successful rope pulling openings across trials in the Sturrell group is shown in Figure 4b.

## Discussion

We found that when presented with an artificial food object that had to be opened by removing a lid from a canister body, three different methods emerged spontaneously in the actions of four vervets, each chosen to act as models in their respective groups. Three of these used their mouths to open the ‘aethipop’ whereas one used only her hands, and one of the three using his mouth also finally used the ropes attached to the lid and canister to pull the two apart. After the opportunity to watch these models, presentation of multiple aethipops to the four groups showed that opening using the mouth was a common approach throughout, but that, importantly, there was a significantly greater preference to manually open the aethipops in the group with the hand-using model and a significant use of the rope-pulling technique developed only in the group with the rope-pulling model.

### (a) Is it imitation?

The results show a clear effect of social learning of the two otherwise relatively rare techniques, manual opening and rope-pulling, in the groups concerned. Following Voelkl and Huber [1], who showed social learning of hand versus mouth techniques for opening a similar artificial food in marmosets, we describe this learning as imitative. We note that Voelkl and Huber went further, referring in the title of their paper to ‘true imitation’, as had Zentall et al. [15] having demonstrated copying of beak versus foot techniques in pigeons (see [22] for a review of action imitation in birds). The rationale of these authors was that such body-part copying clearly differentiates this form of social learning from a principal alternative, emulation, in which subjects reproduce only the environmental results of actions they witnessed, rather than the form of the actions themselves [23]. It is this body-part matching that we have now demonstrated in vervet monkeys, in the mouth-versus-hand contrast. There are still few demonstrations of such body-part copying in animals. It is clear in ‘do-as-I-do’ studies, in which chimpanzees [24] and an orangutan [25] first learned to match a series of training actions on request, and then showed they could copy a significant proportion of novel acts including facial and manual gestures, that cannot be learned by emulation. Bodily copying has most recently been recorded in the context of ‘rational imitation’ in chimpanzees [17] and dogs [26]. In these contexts imitation occurred only when the model appeared to freely choose to use the body part concerned, as opposed to being constrained to use it (e.g. using the head or foot because the hands were already occupied by holding something).

Our interpretation of our results as demonstrating bodily imitation in vervet monkeys is accordingly consistent with this small but growing literature. However, it is important to recognize that the criteria used to define imitation vary much across the social learning literature. One additional criterion relevant to the present results and adopted by several authors in the animal social learning literature is that the imitator learns something new. Thorpe [27], for example, defined imitation as “the copying of a novel or otherwise improbable act or utterance”. We discuss this further below, but note first that this criterion is not considered important in much of the developmental literature (for example, touching the same ear as a demonstrator is classed as imitation in ref [28]) and neuroscience literature (e.g. raising the same finger as a model is the imitation task in [29]).

However, elaborating on Thorpe's criterion, Byrne [30] advocated a distinction between ‘contextual imitation’, in which a behaviour pattern already in the repertoire is applied to a novel context, and ‘production imitation’, in which the novelty resides within the action itself (which could include combining familiar actions into a new and more complex action sequence). It could thus be argued that all the above examples of bodily imitation are only contextual imitation: for example, chimpanzees already know how to press things with their hand or feet; stepping and pecking are already in pigeons' repertoire; the same is true for dogs using their paw or mouth to pull things; and the vervets we studied commonly use both hands and mouth in processing food objects. According to this view, what our vervets learned that was new was the context to which an existing behaviour pattern could be productively applied: what they learn from the model is that part of their existing repertoire (oral or manual) can be applied to gain food from this new aethipop object.

However, although it seems clear conceptually [31], the contextual/production distinction may not be so clear-cut in practice, in large part because novelty itself is not all-or-none [23]; it is very challenging to measure, and in any case relatively novel actions are often constructed on the foundations provided by existing actions. From this perspective, what observer vervets may be learning from the model is *how* to adapt an existing part of their manual or oral repertoire to tackle the novel aethipop object. If so, production imitation would be taking place, perhaps as well as contextual imitation. We suggest that the present results do not allow the contextual/production distinction to be clearly applied and the same is perhaps true of the other cases of bodily imitation by pigeons and marmosets cited above. In any case, it is important to underline that in all our different experimental conditions the monkeys learned about the same environmental result, of getting the tops off the aethipops; what was copied differentially according to the model seen was the production of oral versus manual approaches.

One way to directly approach assessment of the novelty issue is to compare the frequency with which observers later employ an action when they have, or have not, witnessed a model use it. The latter may provide baseline frequencies indicating just how ‘improbable’ the target actions normally are. Thus, Zentall et al. [15] showed that pigeons who saw a model step on a treadle to gain food rather than peck it, never themselves pecked it, whereas if pigeons did witness pecking the treadle rather than stepping on it they had a 0.5 probability of pecking it. Accordingly the pecking was in Thorpe's [27] terms ‘improbable’ in this context, despite the fact that ‘pecking’ is broadly within pigeons' repertoire, and the authors described the copying effect as true imitation. Voelkl and Huber [1] noted that their marmosets would naturally use only their hands to open canisters, and so trained an alternative model to use its mouth, which when matched by four out of six observers thus counted as a novel response in this context and was classed as imitation. Our results are subject to a similar logic, insofar as the hand-opening used by monkeys in the group with a hand-opening model was never seen to be successfully used in the other three groups and was to this extent improbable in this context. Because this also involves bodily matching we accordingly describe it as bodily imitation.

Of course this does not imply that the differences generated in this way would be sustained over a longer timeframe. We note that manual attempts were made by two monkeys in groups other than the one with the hand model, so that over a more sustained period manual successes might become more common than recorded here. We address this further in the section on spread of the different techniques (d), below. However, we note that we had a database of 150 aethipop openings through which to estimate rarity of target actions in the groups without the relevant models (i.e. 0/150 for hand successes, 0/150 for rope successes).

In the case of the rope-pulling technique, there is additional scope for emulation learning of this environmental affordance (e.g. learning that ‘rope-and-lid will separate from rope-and-canister’). However even in this case it remains possible that what was copied from the model was something more like a complex of action-and-result. In chimpanzees, ‘ghost’ experiments in which only the end results of actions are visible have shown that learning fails, by contrast with a condition in which the whole model-action-result scenario has been witnessed [32]. Such experiments could be instructive in exploring the learning processes involved in the acquisition of rope-pulling.

### (b) Immediacy versus delay in matching

We noted that copying occurred significantly on first trials only in the case of hand-opening; it emerged only later in the rope-pulling group. There are perhaps two plausible, potential explanations for this difference. One is that the hand model was a dominant female, the category that earlier field experiments identified as the most influential class of models amongst (wild) vervets [21] whereas we were constrained to use only an adolescent male in the group that developed rope-pulling. The other explanation is simply that this male produced only one example of rope-pulling when acting as the initial model. It may have a spread to others who had already been prone to use their mouths in their first contact, after the adult male adopted rope-pulling during the third trial with 10 aethipops, but there are insufficient data to rigorously assess this, nor to distinguish between the two explanations (model identity versus frequency of demonstrations). However, such potential effects would be amenable to further systematic experimental investigation.

### (c) A role for mirror neurons?

When Voelkl and Huber [1] reported their marmoset bodily imitation results, research on mirror neurons was still in its infancy. Now it has become a well-established field of research to which the present results have potential relevance, because the core characteristic of mirror neurons as originally identified in macaque monkeys is to fire both when the self is performing a goal-directed act such as grasping an object, and when one sees another individual do the same thing. In relation to the present results, we note that such neurons have been identified for both manual actions [33] and oral actions [34] (see [35]–[36] for reviews). As Keysers recently noted [37], a lack of evidence for imitative social learning in monkeys meant that functions other than imitation were attributed to them. However increasing evidence for imitative or other mirroring/matching forms of social learning in primates suggests this was perhaps premature. There is evidence that mirror neurons are involved in imitation in humans [38] and their functional properties correspond well with the bodily matching documented here. We suggest there could be a potential role for the mirror neuron system in supporting the kinds of social learning we have described. Keysers [39] has noted that in the monkey brain, mirror neurons classed as “broadly congruent”, respond equally to a goal being achieved by such different actions as mouth or hand and are about twice as common as those that are “strictly congruent” to a specific action like manually opening a lid. He thus predicts that the major effect of observation on later action should be expected to be emulative, but that the presence of about 30% strictly congruent mirror neurons could support a weaker facility in copying the particular observed means. Our results fit this conception, with limited evidence of bodily imitation existing against a background of more dominant emulation: we found significant evidence of imitation in the group with the model who opened the aethipops with her hand, yet the most common response was an emulative, oral one. However, these considerations are of course speculative: we provide no direct evidence here for the involvement of mirror neurons in the social learning we document.

### (d) Social learning and the spread of innovations

Finally, we address the third part of the title of our paper, which refers to the ‘spread’ of foraging techniques, and thus connects with the growing literature on primate traditions and culture [40]. Clearly, we can make only the most modest of claims on this issue, for our experiment was limited to just five trials over a short period. Nevertheless a novel aspect of our study in comparison to the others on bodily imitation cited above is that we linked the methods to an ‘open diffusion’ design in which we could document any spread of the rarer actions from the initial model to others. As we have seen, such spread occurred in the case of hand use in H group and rope-pulling in S group, as documented in our principal analyses. Given vervets habitually use both their hands and mouth in foraging on items like the artificial ones we presented, with the mouth preferred in this case, we would not expect the manual versus oral technique differences between groups to become robust traditions and indeed, the effect appeared to attenuate already in our study, for the oral/manual contrast (Fig. 4a). However, it is worth noting that in a recent field study of traditions among spider monkeys, the authors described differences in the tendency to use the hands versus the mouth in the different groups studied [41]: such conformity, although surprising, may be more common than we have appreciated. In the case of rope-pulling we did not observe attenuation (Fig. 4b), but rather, signs of continued spread of this technique. Unfortunately, release of the vervets we studied back into the wild means that the potential spread of these techniques cannot be studied in the longer term in this case. However, our results encourage us to extend the approach begun here to wild vervets, with potential for just such longer-term study, extending our existing corpus of field research on this topic [21], [42], [43].
